# Comparative transcriptome analysis of brain and gonad reveals reproduction-related miRNAs in the giant prawn, *Macrobrachium rosenbergii*

**DOI:** 10.3389/fgene.2022.990677

**Published:** 2022-08-26

**Authors:** Jiao Xia, Dong Liu, Wenzong Zhou, Shaokui Yi, Xinhai Wang, Beilei Li, Muhammad Jawad, Haijing Xu, Lang Gui, Mingyou Li

**Affiliations:** ^1^ Key Laboratory of Integrated Rice-fish Farming, Ministry of Agriculture and Rural Affairs, Shanghai Ocean University, Shanghai, China; ^2^ Key Laboratory of Exploration and Utilization of Aquatic Genetic Resources, Ministry of Education, Shanghai Ocean University, Shanghai, China; ^3^ Institute of Eco-Environmental Protection, Shanghai Academy of Agricultural Sciences, Shanghai, China; ^4^ College of Life Sciences, Huzhou University, Zhejiang, China; ^5^ Suqian Institute of Agricultural Sciences, Jiangsu Academy of Agricultural Sciences, Suqian, China; ^6^ Huzhou Fengshengwan Aquatic Seed Industry Co. Ltd., Zhejiang, China

**Keywords:** macrobrachium rosenbergii, brain, gonad, RNA-seq, miRNA, reproduction, MiR-133, 5-HT1

## Abstract

*Macrobrachium rosenbergii (M. rosenbergii)*, as a species of common prawn, is a delicacy that is consumed all over the world. By interacting with the target gene 3′-untranslated region (3'-UTR), microRNAs (miRNAs) regulate its expression and ultimately participate in the regulation of reproductive development. However, research focusing on miRNA regulation during gonadal development in *M. rosenbergii* received very little attention. To explore the association between miRNA and reproduction, we performed RNA sequencing (RNA-seq) on brain and gonad organs in male and female *M. rosenbergii*. A total of 494 miRNAs were obtained in RNA-seq, including 31 and 59 differentially expressed (DE) miRNAs in the brain and gonads, respectively. Furthermore, 9 DE miRNAs were randomly selected from the brain and gonads, and qRT-PCR was conducted to validate the results of RNA-seq. Interestingly, dpu-miR-133 was found to be substantially expressed in the male brain and testis but poorly expressed in the female brain, ovary, and other organs. Analysis of dpu-miR-133 by Targetscan and MiRanda predicted to target *5-HT1*. Furthermore, the dual-luciferase reporter assay manifested that dpu-miR-133 can combine with *5-HT1*. Overall, our research work provides basic data for further study on the miRNA-mediated regulation of brain, gonad, and reproductive development of study *M. rosenbergii*.

## 1 Highlights

RNA sequencing was performed and analyzed on the adult brain and gonads of *M. rosenbergii.*
In total, 31 and 59 differentially expressed miRNAs were identified from the brain and gonad of *M. rosenbergii*, respectively.
Dual-luciferase reporter assay proved that a testis biased expressed miRNA-133 targeting *5-HT1.*

## 2 Introduction

MicroRNAs (miRNAs) are small, non-coding, single-stranded RNA molecules that are produced by transcription from intragenic or intergenic genomic sequences. miRNAs are first transcribed into primary miRNA, which is then cut by Drosha enzyme to hairpin precursor miRNA, and finally in the cytoplasm, mature miRNA is cleaved with the help of Dicer enzyme (O'Brien et al., 2018). miRNAs as considered key post-transcriptional regulators, specifically target the 3'-UTR of mRNA to cleave it or to restrict translation, which in turn regulates several physiological and developmental events (Bartel, 2004; Brennecke et al., 2005). Interestingly, each mRNA has recognition sequences for many miRNAs, and a single miRNA can target thousands of them (Miranda et al., 2006; Zhu et al., 2015). miRNAs are involved to regulate several aspects of life, such as cell communication, metabolism regulation, and cell differentiation (Betel et al., 2008). Furthermore, recent studies indicate that miRNAs are essential for the germ cell differentiation and the development of the reproductive system (He et al., 2012; Hossain et al., 2012; Meng et al., 2018; Zou et al., 2020).

Gamete growth as well as production in the gonads is a prerequisite for reproduction, which entails an intricate and exactly harmonized process, consisting of changes in the morphology and function of different kinds of follicles or spermatogenesis cells (Piprek, 2010). In order to control mechanisms, the expression of many DEGs must be strictly regulated at the transcriptional and post-transcriptional stages. In *Decapod Crustaceans*, numerous research has been carried out on transcriptional control of gonadal development as well as gametophyte occurrence (Uawisetwathana et al., 2011; He et al., 2012; Qiao et al., 2012; Meng et al., 2015; Peng et al., 2015). Being the most significant post-transcriptional regulator, there has widespread attention on miRNAs in the field of reproduction in recent years. There is growing evidence that miRNAs are vital for all stages of reproductive development, consisting of follicular genesis, spermatogenesis, and steroid production (Macias et al., 2009; Hossain et al., 2012). Biogenesis of miRNAs was inhibited during the initial phases of proliferation and differentiation, which led to cause problems with the maturation of oocytes and disruption of spermatogenesis (Murchison et al., 2007; Hayashi et al., 2008). Currently, miRNAs related to gonads are found in *Eriocheir sinensis* and *Japanese penaeus* (Song et al., 2014; He et al., 2015).

Apart from the gonads, the brain which is a part of the central nervous system is also concerned with the development of the gonads. Discrepancies in the expression of brain genes may lead to sex development differences (Dennis, 2004; Davies and Wilkinson, 2006). So, brain events are likely to regulate the development of the gonads (Francis, 1992). Recent studies have shown that nearly half of the known miRNAs are expressed in mammalian brains (Krichevsky et al., 2003; Landgraf et al., 2007). In *Paralichthys olivaceus*, transcriptome analyses find that there are differential gender bias genes in female and male brains, and nearly half of the genes are targeted by the DEMs during the gonadal differentiation (Zou et al., 2020). So far, information on the role of miRNAs is limited in gonadal differentiation. In short, it is very important to study the miRNAs and target genes in the brain.

The main mechanisms of sex determination in crustaceans are genotype, environmental, and genotype sex determination influenced by the environment (Vogt, 2018), Although there is no direct evidence to confirm the existence of sex chromosome in *M. rosenbergii*, it can be inferred from previous studies that *M. rosenbergii* belongs to WZ/ZZ chromosome sex determination type (Staelens et al., 2008). It is noteworthy that the Androgenic gland hormone (AGH) secreted by the Androgenic gland in *M. rosenbergii* is not only a sex-determining factor but also a regulatory factor of male sex differentiation. Sex-related genes of *M. rosenbergii* have been screened by cDNA library construction and transcriptome sequencings, such as *MR-IAG*(Ventura et al., 2009), *MR-IR* (Sharabi et al., 2016), *Mr-Mrr* (Phoungpetchara et al., 2012) and *MRPINK*(Cao et al., 2007). Researchers have conducted transcriptome sequencing analysis of ovary and testis from18 *M. rosenbergii* based on 454 sequencing platforms and obtained more than 750,000 high-quality clean reads and 44,407 Contigs. Among them, 112 and 270 genes were differentially expressed in ovary and testis of *M. rosenbergii*, respectively, suggesting that these genes may be related to sex differentiation (Jung et al., 2016).

*Macrobrachium rosenbergii* (*M. rosenbergii*) is one of the largest freshwater prawn species that live in both fresh and saltwater. Breeding of this species originated in Malaysia in the early 1960s and is now widely cultivated in South and Western Pacific and Southeast Asia (Bonami and Widada, 2011; Jiang and Qiu, 2013). The growth rate of female shrimps decreases due to the need to provide a large number of nutrients for ovarian development, while the male shrimps can keep growing faster all the time. Therefore, under the same cultural conditions, the average body weight of male shrimps is about twice that of female shrimps. While improvements in productivity and technology have led to huge advances, many development problems have also emerged due to inbreeding and bacterial contamination in breeding and farming techniques. Therefore, it is necessary to conduct in-depth research on the gonadal development mechanism of *M. rosenbergii* and provide a basis for breeding and related technologies optimization.

In this study, for RNA-seq, we collected the brain and gonadal tissues of male and female *M. rosenbergii* individuals, and the results were verified by qRT-PCR. Results obtained from Targetscan and MiRanda predicted the targeting relationship between dpu-miR-133 and *5-HT1*. Furthermore, the dual-luciferase reporter gene test confirmed that dpu-miR-133 could bind to *5-HT1*. These results are helpful for further research into the miRNA-mediated regulation of *M. rosenbergii* brain, gonad, and reproduction.

## 3 Materials and methods

### 3.1 Animals and sample collection

*M. rosenbergii* in good health were collected from Huzhou Fengsheng Bay Aquatic Products Co., LTD. and shipped back to our lab. These prawns were reared in a water circulation system at a temperature of 26°C with 14 h of light and 10 h of dark light on the campus of Shanghai Ocean University (Lin’gang, Shanghai, China). At the time of sampling, *M. rosenbergii* were four months old and had reached the stage of sexual differentiation. After dissection, samples of brain, gonads, muscles, liver, intestines, heart, and gills from both female and male shrimp are frozen in liquid nitrogen at once and kept at −80°C. Research in *M. rosenbergii* was conducted strictly in accordance with the regulations of the Ethics Committee for Experimental Animal Research of Shanghai Ocean University.

### 3.3 Construction of small RNA library

By using TRizol^®^ (Invitrogen, Carlsbad, CA), total RNA was isolated from the brain and gonad of *M. rosenbergii*. Verification of RNA integrity by Agilent 2,100 Bioanalyzer (Agilent Technologies, United States). Small RNA libraries were prepared according to the TruSeq Small RNA Sample Prep Kit (Illumina, San Diego, United States) using 1 μg total RNA. The four libraries were then sequenced using Illumina Solexa technology at OE Biotech. Co., Ltd. (Shanghai, China).

### 3.4 Analysis of sequencing data and miRNAs identification

The data processing is carried out following the described procedure (Yue et al., 2016). Screen the original readings by wiping out low-mass sequences, splicing common RNA families (rRNA, tRNA, snRNA, snoRNA), and repeating sequences. Then we applied strict principles to identify conservative and new miRNAs, consisting of sequences between 18 and 26 nt in length with >10 reads. In addition, conserved miRNAs did not allow seed sequence mismatch, with any more than two mismatches at other locations. All new miRNAs had a similar stem-like structure to that of typical miRNA precursors. The RNAfold software (http://rna.tbi.univie.ac.at//cgi-bin/RNAWebSuite/RNAfold.cgi) was used for the prediction of the secondary structure of new sequences. miRNAs readings were then standardized for better comparisons, assessing differences in MiRNAs between males and females. Target genes were determined by TargetScan and Miranda software to better understand the differentially expressed miRNAs. Then we use GO and KEGG enrichment analyses.

### 3.5 miRNA Extraction and qRT-PCR

The PrimeScript™ First Strand cDNA Synthesis Kit (Takara, Shiga, Japan) was used to reverse transcribe total RNA into cDNA. Using Primer Premier 6.0 to design the divergence primer (Supplementary Table S1). The miRNA was subjected to a reverse transcription PCR (RT-PCR) chain reaction. The reaction mixture is made up of a 1 μL cDNA template, 0.5 μL reverse primers 10 mm, and 18 μL premixed Taq (Takara Taq version2.0 + dye). A total of 35 cycles, each containing 95°C denatured 15 s, 60°C annealed 15 s, and 72°C extended 15 s, were used to obtain PCR product agarose gel DNA extraction kit (Takara, Shiga, Japan) from a 1.5% agarose gel using MiniBEST, and further verified by Sanger sequencing. To study the expression of miRNAs in *M. rosenbergii*, 9 DE miRNAs in the brain and gonad were randomly selected. Using ABI7500 real-time fluorescence quantitative polymerase chain reaction system and TB Green^®^PreMix Ex Taq™II (Japan), we verified the tissue distribution of nine miRNAs in the muscle, liver, gill, heart, intestine, both male and female-brain, ovary, and testis of the adult of *M. rosenbergii*. The qRT-PCR amplification procedure is consistent with previous descriptions (Sun et al., 2020). Following amplification, the products were evaluated using dissociation curves. With *U6* as the endogenous reference gene, the expression level was normalized by the 2^−ΔΔCt^ method. IBM SPSS Statistics 25.0 software was used to test the association between qRT-PCR and RNA-seq.

### 3.6 Prediction of miR-133 targeting gene for *5-HT1*

Depending on seed region pairing and minimal free energy, miR-133 probable targets were predicted using TargetScan (Lewis et al., 2003) and bioinformatics analysis of RNA22 v2 (http://www.mybiosoftware.com/).

### 3.7 Dual-luciferase reporter assay

miR-133 has a binding site to *5-HT1* 3'-UTR. To validate miR-133 targets, a luciferase reporter plasmid was constructed. Clone a miR-133 binding sequence containing 5-HT1 3'-UTR and insert the pmirGLO vector. By mutating the predicted miR-133 seed region, a 5-*HT1* 3'-UTR mutant reporter gene was cloned by PCR, and the primers were shown in Supplementary Table S1. For *5-HT1* mutant, the binding sequence (CCG​GTT​GAG​AAG​GAC​CAA​ATG​CAG​TTA) was replaced by CCG​CAT​GTG​AAG​CTG​CAA​ATG​CAG​TTA using the primers of *5-HT1*-MT-FW and *5-HT1*-MT-RV, which was conducted into pmirGLO vector through XbaI and SalI sites. The plasmids were sequence-verified. The culture and transfection methods of human embryonic kidney 293 (HEK293) cells used in the experiment were as previously described (Sun et al., 2020). HEK293 cells were co-transfected with wild-type (WT) or mutant-type (MT) plasmids and miR-133 mimics (GenePharma, China) using FuGENE^®^HD (Promega, United States). The Dual-Glo^®^ Luciferase Assay System (Promega, United States) was used to quantify the luciferase activity 36 h after transfection. At least three times each experiment was conducted.

## 4 Result

### 4.1 Characterization of gonadal small RNA

In this research, using a sample of ovary, testis, and brain of both gender, four libraries were created from small RNA. Initially, the four libraries generated raw reads, 32.84, 47.58, 28.63, and 29.78 M. After removing low-quality sequences, it had 22.18 M, 27.25 M, 24,49 M, and 24.65 M clean reads were included in the relevant libraries. In the library, non-coding RNAs are categorized based on their sequence similarity to short RNA sequences in Rfam: rRNA, snRNA, tRNA, known miRNA, and un-annotated. (Table 1). To identify miRNAs in the brain and gonads of M. rosenbergii, we compared the sequences of the brain and gonads of M. rosenbergii with that of mature miRNAs in miRBase21.0, resulting in 271 conserved miRNAs and 223 new miRNAs (Table 2). In this study, we found dpu-miR-100 with 3,416,104 reads in the female brain and dpu-miR-1 with 775,001 reads in the testis of males were the most abundant and conservative miRNAs. In addition, tcf-miR-184-3p, dpu-miR-71, and dpu-miR-276 were captured over a hundred thousand times. Furthermore, the box beard plot was used to understand the distribution of miRNA in the four libraries (Supplementary Figure S1).

**TABLE 1:** Reads distribution of four small RNA libraries. Note: FB, female brain; MB, male brain; O, ovary; T, testis.

Category	FB	MB	O	T
	Reads	Reads	Reads	Reads
rRNA	28104	59901	16446	17251
tRNA	8522	17424	3600	3744
snRNA	8949	9364	5120	7315
miRNA	5196477	6884909	3395349	4705874
annotation	14665915	16807200	17034140	15853687

**TABLE 2:** Statistics of known and novel miRNAs in four small RNA libraries. Note: FB, female brain; MB, male brain; O, ovary; T, testis.

Library	Known miRNA	Novel miRNA
FB	68	56
MB	68	62
FG	66	61
MG	69	44

### 4.2 Distribution of miRNA length

**FIGURE 1:**
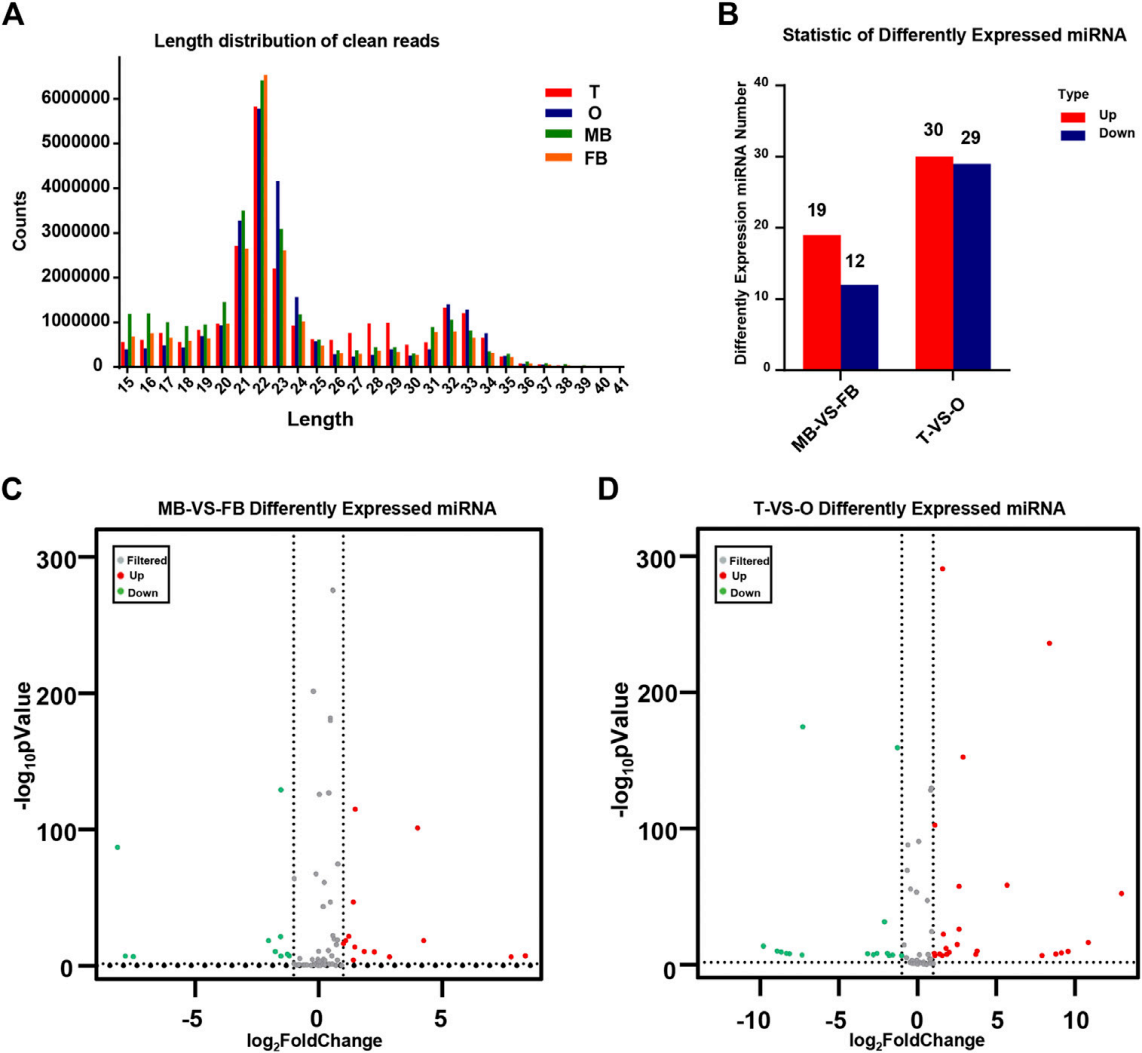
Identification of miRNAs. **(A)** The distribution of miRNA length from the *M. rosenbergii.* **(B)** Statistic of differently expressed miRNA. **(C)** Volcanic map of *M. rosenbergii* differences in the brain. **(D)** Volcanic map of *M. rosenbergii* differences in the gonad. MB: male brain; FB: female brain; T: testis; O: ovary.

The distribution of miRNA length presents a distinctly different bimodal pattern in terms. According to an analysis of the length distribution, 22 nt was the main size, and 21 and 23 nt is followed, which is the same as the typical characteristics of Disher enzymes. Another peak for 32 nt mainly represents endogenous small non-coding RNA molecules, called longer piwi-interacting RNAs (piRNAs). As shown, the peak represented by the 21–23 nt size class was much higher than the peaks of the longest and shortest size class, indicating the abundance of small RNAs in the brain and gonad of *M. rosenbergii* (Figure 1A).

### 4.3 Target gene prediction and bioinformatic analysis

By comparing the expression of miRNAs in various libraries, we were able to obtain DE miRNAs. On the other hand, comparing the DE miRNAs between the female and male brain, 31 mature miRNAs with remarkable up-or down-regulation in the female brain as compared to the male brain, including 19 female brain-biased and 12 male brain-biased miRNAs. Furthermore, comparing the DE miRNAs between ovaries and testis, 59 miRNAs were found in total, of which 29 miRNAs in the ovary were higher than that in testis, while 30 miRNAs expression was lower in the ovary (Figure 1B). The volcanic map shows significant DEMs in the brains and gonads of males and females (Figures 1C,D).

**FIGURE 2:**
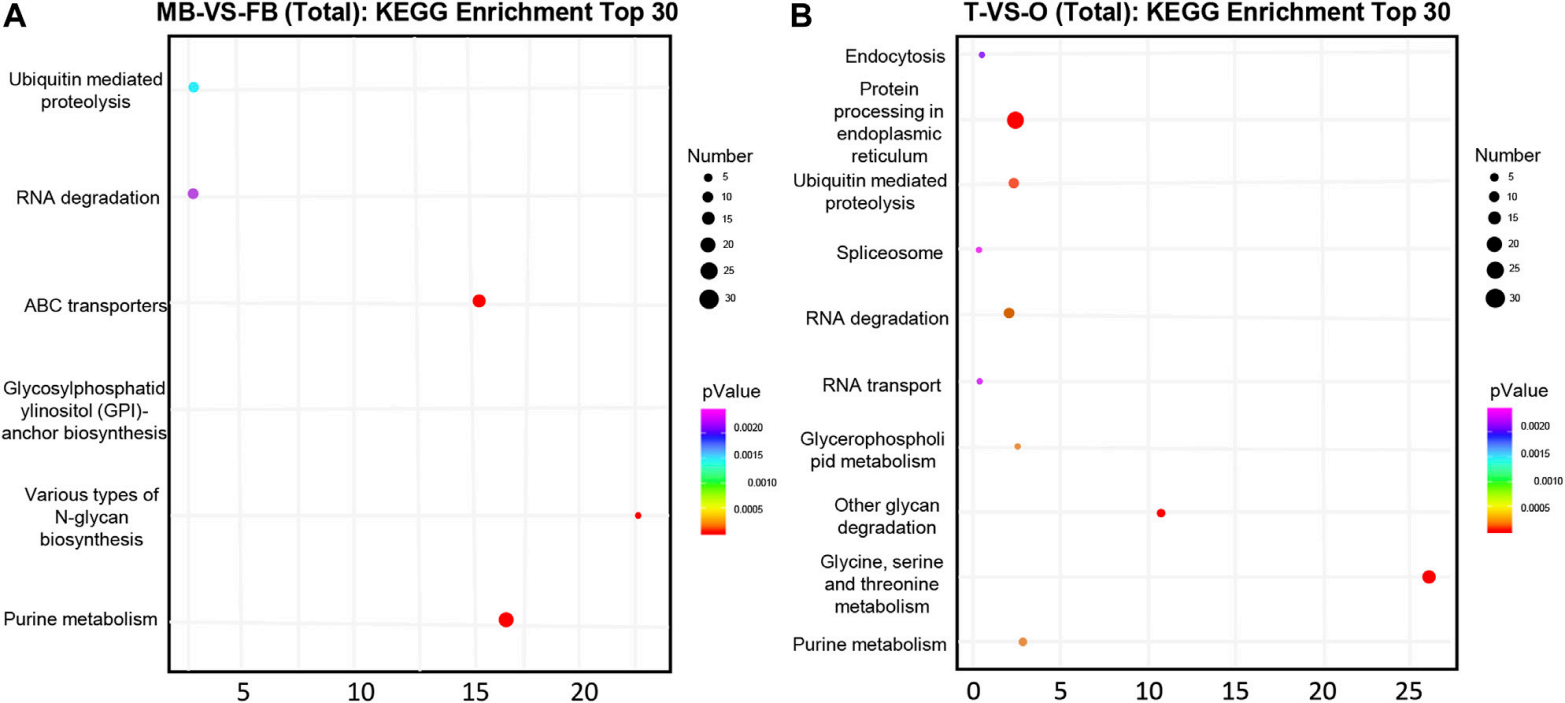
Top 30 significantly enriched KEGG pathways of putative target genes between the female and male *macrobrachium rosenbergii* in the brain **(A)** and gonad **(B).** Significant enrichment of the KEGG pathway (*p* < 0.05). The color represents the size of the *p*-value and the point represents pathway. The redder the color, the smaller the *p*-value, the larger the number of points, and the more enriched genes. MB: male brain; FB: female brain; T: testis; O: ovary.

Overall, 116 and 1,255 genes were considered potential targets for expression levels of miRNAs in the brain and gonad, respectively. Most DE miRNAs have multiple target genes, and most of them are regulated by multiple miRNAs. We conducted an enrichment analysis of GO as well as KEGG, to identify the biological role of the DE mRNAs. In KEGG pathway analysis, we set the *p*-value at 0.05 as the threshold. As compared male brain VS. female brain, several signaling pathways, such as purine metabolism, glycosylphosphatidylinositol, and various types of N-glycan biosynthesis were enrichment (Figure 2A). While in the gonads, Ubiquitin mediated proteolysis and protein processing in the endoplasmic reticulum, Glycine, Serine, and threonine metabolism were also involved in its development (Figure 2B).

**FIGURE 3:**
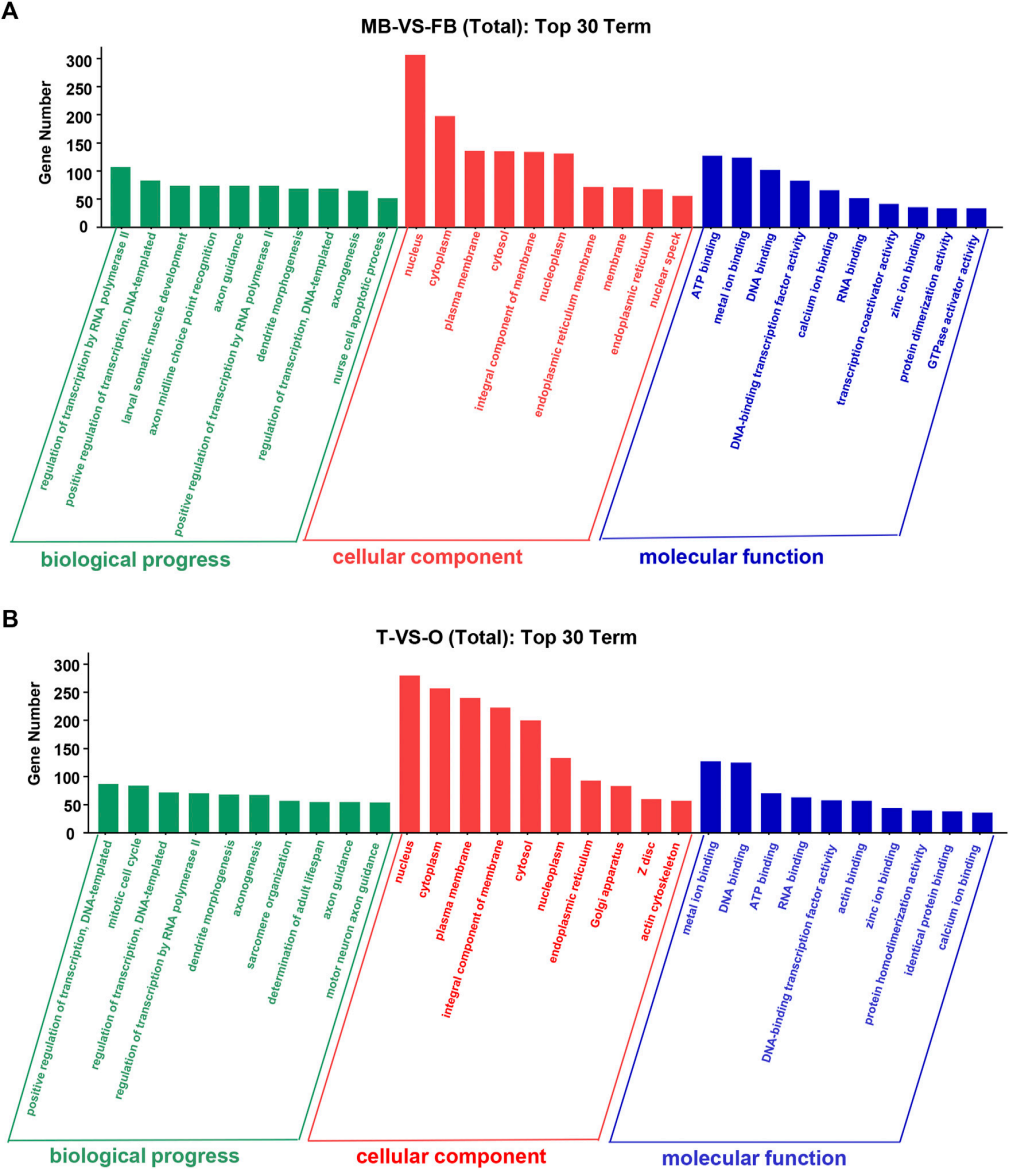
Top 30 GO terms of unique genes targeted by DEMs from the female and male *macrobrachium rosenbergii* in the brain **(A)** and gonad **(B)**. GO analysis falls into three categories: biological processes, molecular functions, and cellular components. MB: male brain; FB: female brain; T: testis; O: ovary.

At level 2, GO categories are divided into three groups of potential. In the three categories of terms of male brain VS. female brain, the most abundant GO terms are “regulation of transcription by RNA polymerase II”, “nucleus”, “ATP binding”, and “metal ion binding” (Figure 3A). The GO terms such as “positive regulation of transcription”, “DNA-templated nucleus”, “metal ion binding”, and “DNA binding” were rich in the ovary as compared to the testis (Figure 3B).

### 4.4 qRT-PCR verifies DEMs

**FIGURE 4:**
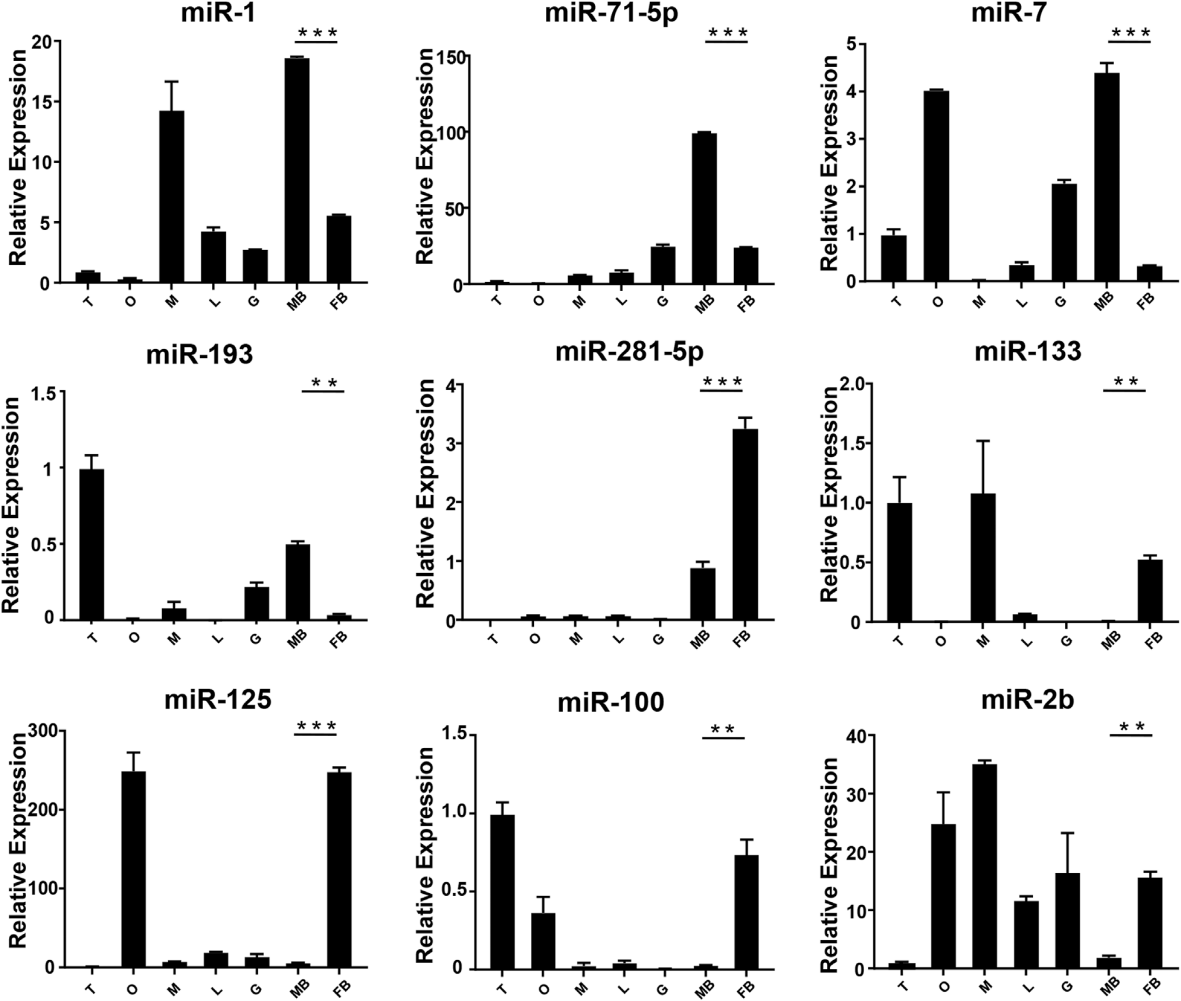
The expression of DEMs in the brain of *M. rosenbergii*. The endogenous reference gene was *U6*, and its expression was determined by comparative CT (^ΔΔCT^) methods. T: testis; O: ovary; M: muscle; L: liver; G: gill; MB: male brain; FB: female brain. *: 0.01 < *p* < 0.05; **: 0.001 < *p* < 0.01; ***: *p* < 0.001.

**FIGURE 5:**
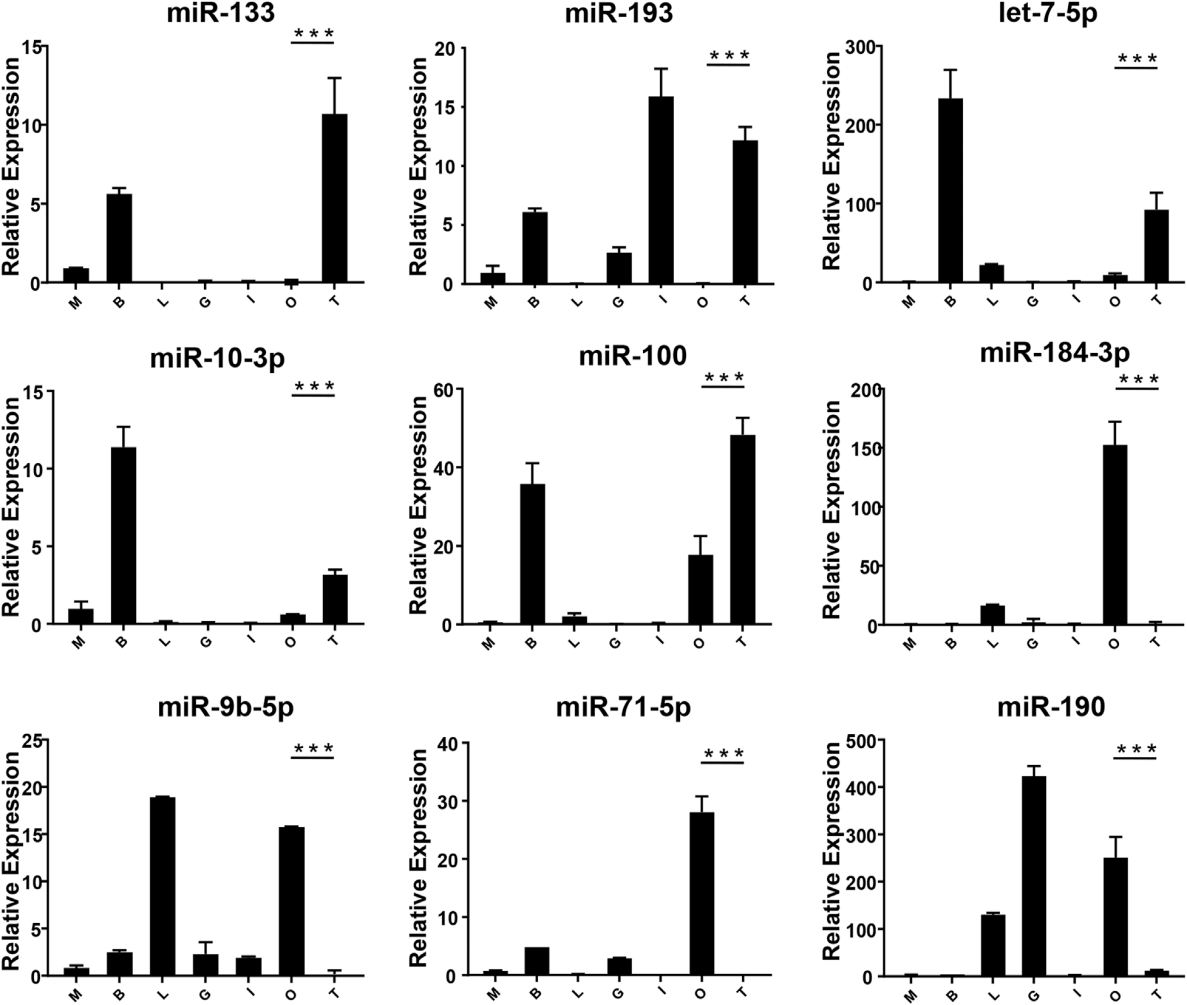
The expression of DEMs in the gonad of *M. rosenbergii*. The endogenous reference gene was *U6*, and its expression was determined by comparative CT (^ΔΔCT^) methods. M: muscle; B: brain; L: liver; G gill; I: intestines; O: ovary; T: testis. *: 0.01 < *p* < 0.05; **: 0.001 < *p* < 0.01; ***: *p* < 0.001.

In order to verify the expression of DE miRNAs in *M. rosenbergii*, 9 DE miRNAs were randomly selected from the brain and gonad and performed qRT-PCR to verify their distribution in the eye, brain, muscle, liver, gut, ovary, and testis for further investigation (Figures 4,5). The relative expression of *U6* single-stranded RNA was measured as the endogenous reference gene, and the relative expression of DE miRNAs screened out was consistent with the sequencing results. In general, DE miRNAs expression was significantly different in the brain and gonad of both genders of *M. rosenbergii.*

### 4.5 *5-HT1* is Regulated by miR-133

**FIGURE 6:**
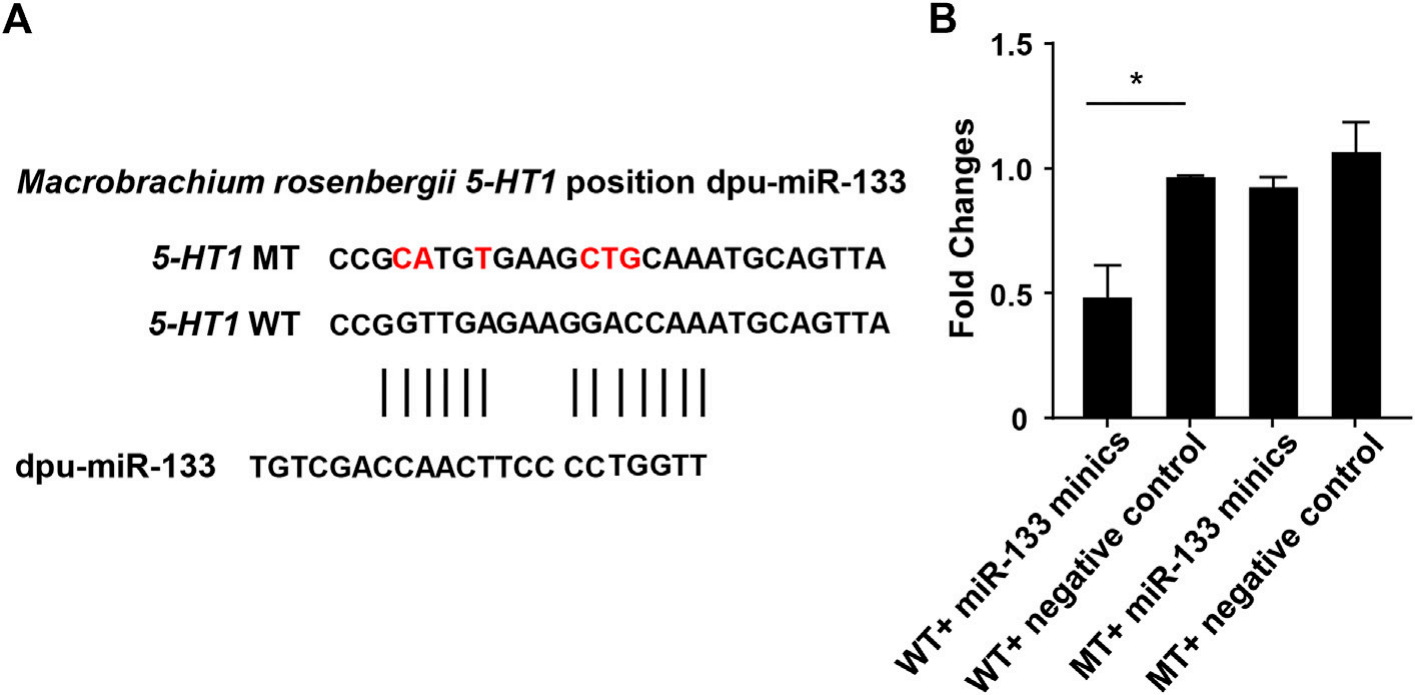
Double luciferase reporter gene analysis of miR-133 and *5-HT1*. **(A)**. The binding site of miR-133 to *5-HT1* 3’-UTR. **(B)**. Relative luciferase activity in HEK293 cells. GraphPad Prism 7 draws a histogram. The means ± standard deviations of the three independent experiments are shown in the data. *p < 0.05.

Through the above software analysis, we found that miR-133 targets the *5-HT1*-3'UTR conservatively (Figure 6A). To further validate the targeting relationship between *5-HT1* and miR-133 *in vitro*, the pmirGLO -*5-HT1*-3'UTR and miR-133 mimics were co-transfected into the HEK293 cells. The results confirmed that expression of *5-HT1* was significantly inhibited under the regulation of miR-33, while there were no differences between mutant-type (MT) and negative controls. (Figure 6B). The findings strongly imply that *5-HT1* potentially regulates the development of gonads and gamete generation in *M. rosenbergii.*

## 5 Discussion

Even though miRNAs are crucial for controlling gene expression throughout gonadal development and sexual differentiation (Qiu et al., 2018; Sun et al., 2020), Up till now, little research has reported the involvement of miRNAs in crustacean reproductive development (He et al., 2012; Song et al., 2014). Therefore, this research aimed to find DEMs in *M. rosenbergii* and to assess differential expression patterns in the brain and gonad.

In current research work, 494 miRNAs were detected totally in the brain and gonads of *M. rosenbergii*, including 271 known miRNAs and 223 novel miRNAs, contributing to the limited database of miRNAs in crustaceans. In general, the abundance of novel miRNAs is lower than that of conserved miRNAs, suggesting that novel miRNAs may play a role or be of lower functional importance in specific gonad stages (Ro et al., 2007). The distribution analysis of miRNA length populations suggested that the size distribution of all four libraries was similar, with a peak at 22 nt, while only the testis library had a peak at 27–29 nt. Peaks in 21–22 nt length represent representative sizes of Dicer derivatives, while peaks at 27–29 nt may be from piRNAs. Usually, piRNAs are about 25–30 nt in length and have high expression in mice, insects, and fish in the gonads (Grimson et al., 2008; Klattenhoff and Theurkauf, 2008). The sperm of *M. rosenbergii* contained small RNA of similar size (27–29 nt RNA peak), suggesting that piRNA may regulate the development of testis.

Among the 494 miRNAs, we focused on DEMs and identified 49 female-biased miRNAs and 31 male-biased miRNAs. These DEMs might be involved in gender differentiation and gonadal development. For example, miR-7, miR-71, let-7-5p, and miR-184 are necessary for the development of the brain (Diotel et al., 2011). The effect of estrogens, its metabolites, and testosterone have different effects on the development of males’ and females’ brains (Negri-Cesi et al., 2004; He et al., 2012). Previously, researchers found high expression of miR-7 in the pituitary gland of mice and pigs (Li et al., 2011; He et al., 2020). In sows, miR-7 inhibits the synthesis and secretion of *Follicle-Stimulating hormones* (He et al., 2018). Mice knocked out of MiR-7 present with little gonads production and infertility (Ahmed et al., 2017). miR-71 plays a role in the neurological system and mediates the impact of the germ line on lifespan (Boulias and Horvitz, 2012). Our findings demonstrate that the expressions of miR-7 and miR-71 in the brain of the male in *M. rosenbergii* were higher than those in the female, suggesting that they might involve in male reproductive development by regulating brain hormones. Previous studies shows, the wide expression of let-7 family in the gonads of insects, fish, and birds (Bizuayehu et al., 2012; Gu et al., 2014; Quah et al., 2015). In our study, let-7-5p, which is a member of the let-7 family, was highly expressed in the testis as compared to the ovaries, suggesting that let-7-5p may participate in the male reproduction of *M. rosenbergii*. Apart from it, miR-184-3p is expressed highly in the ovary compared to the testis, suggesting that miR-184-3p might participate in the female reproduction of *M. rosenbergii*. In agreement with our findings, recent research has demonstrated that miR-184 plays a role in the female reproductive system development, and the loss of miR-184 may lead to serious defects in *Drosophila* eggs (Iovino et al., 2009). At the same time, miR-184 also plays a role in male reproductive development by contributing to the formation of germ cells (Wu et al., 2011).

miR-133b belongs to the miR-133 family, and the research by predecessors has shown that miR-133b is expressed in both mouse and human oocytes and follicles (Dai et al., 2013; Xiao et al., 2014). After gonadotropin-releasing hormone treatment, miR-133b is up-regulated in porcine pituitary cells, suggesting that it may affect gonadotropin release by targeting gonadotropin β and C-Jun (Ye et al., 2013). In *tilapia*, miR-133b may modulate *tagln2* expression to achieve inhibition of primitive follicle formation (Ma et al., 2021). In crustaceans, similarly, miR-133 is differentially expressed in crab oocytes during meiosis and maturation and regulates the 3'-UTR of the crab *cyclin B* (Song et al., 2014). In this research, the expression of miR-133 in *M. rosenbergii* was shown to be highly expressed in the brain and ovaries of females, and relatively low or no expression in the brain and testis of males. The analysis of dual-luciferase reporter had shown that dpu-miR-133 may down-regulate the expression of *5-HT1* by predicting targets in the 3'-UTR.

As an important biogenic amine, 5-hydroxytryptamine (*5-HT*) is involved in the important physiological processes in decapod crustaceans. The serotonin receptor family is classified into 7 subfamilies (*5-HT1*-*5-HT7*) (Cortes-Altamirano et al., 2018). *5-HT* can induce ovarian maturation in *Penaeus vanamei*, (Vaca and Alfaro, 2000), *Penaeus monodon* (Wongprasert et al., 2006), *Platycephalus indicus* (Tomy et al., 2016), *Procambarus clarkii* (Kulkarni et al., 1992) and *M. rosenbergii* (Soonthornsumrith et al,, 2018). *5-HT* can promote the secretion of estradiol-17β (*E2*) and progesterone (*P4*) in the ovary (Benmansour et al., 2014). We predict that *5-HT* may participate in ovarian maturation by localizing specific brain regions and ovaries of shrimp, inducing the secretion of estrogen, which may promote the development of yolk and thus lead to the growth and maturation of oocytes (Soonthornsumrith et al., 2018).

In conclusion, we reported known and novel miRNAs in the brain and gonads of female and male *M. rosenbergii*. Based on the analysis of miRNAs, 80 DEMs were identified. The analysis of GO and KEGG showed that some DE miRNAs were involved in gonadal development. Through software prediction, miR-133 was found to target *5-HT1*, and its targeting relationship was verified by a dual-luciferin report. The data presented in this research can provide the basis for additional information on the genetic mechanism of *M. rosenbergii* and valuable information for the reproductive control technology of *M. rosenbergii*.

## 6 Institutional review board statement

The current research work was carried out in accordance with the Declaration of Helsinki and the Shanghai Ocean University Animal Care and Use Committee granted consent for the study with the approval code SHOU-2021-118.

## Data Availability

The datasets (ANALYZED) for this study can be found in the Figshare https://doi.org/10.6084/m9.figshare.20388261.v1e.

## Abbreviations

DEM, differentially expressed miRNA; FB, female brain; GO, gene ontology; KEGG, kyoto encyclopedia of genes and genomes; MB, male brain; qRT-PCR, quantitative real-time PCR; nt, nucleotide.
