# Supplementary material for: Comparative transcriptome analysis of brain and gonad reveals reproduction-related miRNAs in the giant prawn, Macrobrachium rosenbergii
Source: Front Genet. 2022 Aug 26;13:990677. doi: 10.3389/fgene.2022.990677 (PMC9459145; doi:10.3389/fgene.2022.990677)
Supplement: Supplementary file 1 [file Table1.DOCX]

Supplementary Material

**Supplementary Table S1.** List of primers in this article.

| Primer name | Primer sequence |
| --- | --- |
| dpu-miR-1 | GCGCGCGTGGAATGTAAAGAAGTATG |
| dpu-miR-7 | GCGCGCTGGAAGACTAGTGATTTTGT |
| tcf-let-7-5p | GCGCGCTGAGGTAGTAGGTTGTATG |
| tcf-miR-9b-5p | GCGCTCTTTGGTGGTCTAGCTGTA |
| tcf-miR-71-5p | GCGCGTGAAAGACATGGGTAGTGA |
| dpu-miR-100 | GCAACCCGTAGATCCGAACTTG |
| dpu-miR-133 | GCTTGGTCCCCTTCAACCAGC |
| dpu-miR-193 | GCGTACTGGCCTGCTAAGTCCC |
| tcf-miR-125 | GCGTCCCTGAGACCCTAACTTG |
| tcf-miR-281-5p | GCGCAAGAGAGCTATCCGTCGAC |
| tcf-miR-2b | GCGTATCACAGCCAGCTTTGAC |
| tcf-miR-184-3p | GCGCGTGGACGGAGAACTGATAAG |
| tcf-miR-10-3p | GCGCCAAATTCGGTTCTAGAGAGG |
| tcf-miR-190 | GCGCGCAGATATGTTTGATATTCTTGG |
| SnRNA | TTGGAACGATACAGAGAAGATTAGCAT |
| *5-HT1*-WT-FW | CCGGTTGAGAAGGACCAAATGCAGTTA |
| *5-HT1*-WT-RV | TAAGTCATCCGTTCGTGTCGTCAAGCG |
| *5-HT1*-MT-FW | CCGCATGTGAAGCTGCAAATGCAGTTA |
| *5-HT1*-MT-RV | TAAGTCATCCGTTCGTGTCGTCAAGCG |


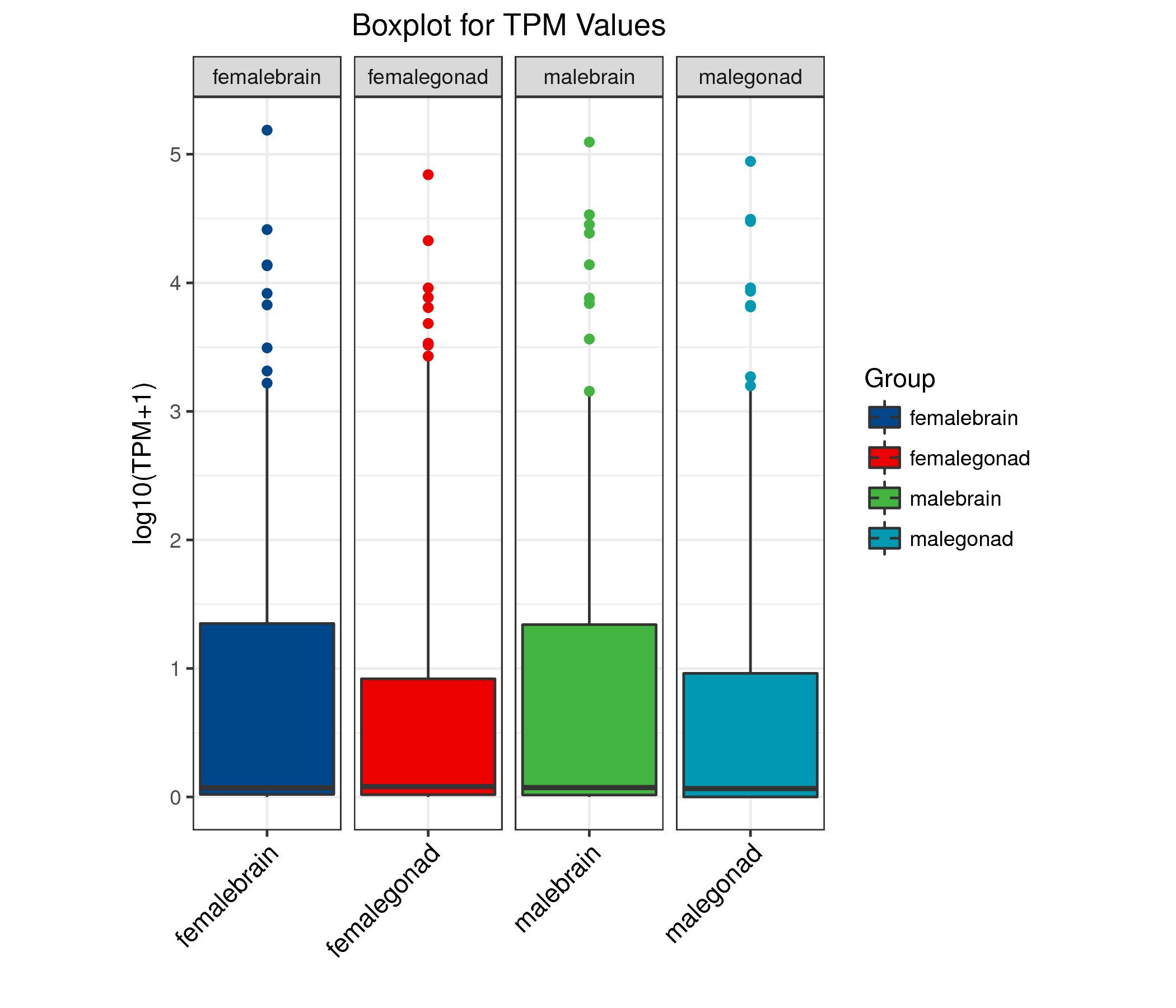


0

2

4

3

1

5

FB

MB

O

T


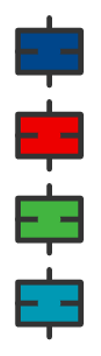


O

MB

T

FB

**Group**

log_10_(TPM+1)

Boxplot for TPM Values

**Supplementary Figure S1.**  Box-whisker plot of miRNA expression among four libraries. MB: male brain; FB: female brain; T: testis; O: ovary.
